# Effect of Post-Process Curing and Washing Time on Mechanical Properties of mSLA Printouts

**DOI:** 10.3390/ma14174856

**Published:** 2021-08-26

**Authors:** Bartłomiej Nowacki, Paweł Kowol, Mateusz Kozioł, Piotr Olesik, Jakub Wieczorek, Krzysztof Wacławiak

**Affiliations:** 1 Faculty of Electrical Engineering, Silesian University of Technology, ul. Bolesława Krzywoustego 2, 44-100 Gliwice, Poland; bartnow218@student.polsl.pl (B.N.); pawel.kowol@polsl.pl (P.K.); 2 Faculty of Materials Engineering, Silesian University of Technology, ul. Krasińskiego 8, 40-019 Katowice, Poland; piotr.olesik@polsl.pl (P.O.); jakub.wieczorek@polsl.pl (J.W.); krzysztof.waclawiak@polsl.pl (K.W.)

**Keywords:** 3D printing, mask stereolithography, post-process, mechanical properties, hardness, profilographometry

## Abstract

The article discusses the influence of the post-process on the mechanical properties of elements produced with the use of the mask stereolithography (mSLA) method. Printed samples were subjected to the following post-process steps: Washing and post-curing, at various times. Then, static tensile and static bending tests were carried out, as well as Shore D hardness measurements for the inner and surface part of the sample, as well as profilographometric analysis of the surface. The post-curing time has been found to strongly affect the tensile and bending strength of printouts, and to improve their surface quality. Washing has an ambiguous effect on the strength of the printouts, but, in the end, it was found that extended washing slightly reduces the strength. Washing significantly affects the quality of the printout surface. A washing time that is too short results in a surface that strongly resembles the printing process, with high roughness. Increasing the washing time to 10 min lowers the roughness by one order of magnitude. Post-curing has also been shown to be beneficial for the cured sample with the application of shielding water. This approach results in an improvement in the flexural strength of the printouts. In general, the obtained research results indicate that, for printouts with cross-sectional dimensions of several mm, the optimal washing time is no more than 10 min and the post-curing time is at least 30 min.

## 1. Introduction

Stereolithography (SLA) is historically the first used additive manufacturing (AM) process [1,2]. This technique is characterized by the smallest number of process parameters. It guarantees an excellent quality of the print surface compared to other AM techniques. It consists in hardening the photopolymer in a liquid state, with the use of ultraviolet (UV) light. The polymerization process consists of twostages. Initially, gelation occurs, which is the process of creating an infinite molecular network [3,4]. Then, the vitrification process begins, i.e., a gradual thermoreversible process of the formation of a glassy material [5,6]. The printed “green” element consists of the following two phases: gel and sol. The printout is anisotropic, due to the additive nature of the process [2,5]. A problem occurs in reaching the inner areas of the printout, by UV rays, which is necessary to cure the sol phase. A properly long exposure time leads to polymerization of unlit areas, due to a chain reaction that is maintained by the large amounts of photoinitiators added to the resins that are used in SLA technology [5]. Initially, a UV laser light source was used for curing the polymers. In recent years, however, devices using light electric diodes (LED) have appeared. Devices of this type use LED matrices to harden the appropriate tracks in the resin, producing a properly pathed layer. This technique is referred to as mask stereolithography (mSLA) [1,2]. This process quantizes the produced object to cuboids, with sides corresponding to the pixel size and the thickness of the hardened layer [1,2,5]. The mSLA technique is used, among others, for printing biomaterials [7], metamaterials for mechanical applications [8], and elements for microelectromechanical systems (MEMS) [9].

The mSLA process requires a post-process that must be carried out after printing is complete, and essentially consists of two steps. First, it is necessary to remove excess adhering uncured resin from the printout. This stage is called washing. It consists of vigorously pouring the printout with a liquid (usually 2-propanol) in a closed chamber. This process is extremely important for maintaining the greatest advantage of SLA prints, which is good surface quality [1,2]. During the washing process, the sol phase is washed out from the subsurface areas of the printout. The next process is the final hardening of the resin, in a process called post-curing. This process is extremely important because it ensures the final mechanical properties of the resin [1,2]. Post-curing time is a parameter that is often neglected by resin producers. A poorly chosen post-curing time can also lead to deformation of the printout. This is due to the shrinkage caused by the resin crosslinking [10]. After a sufficiently long post-curing exposure time, the stresses that occurred inside the material should disappear. Due to the dimensional and geometric limitations during printing, it is the post-curing that significantly affects the final properties of the manufactured detail [1,5,11].

The post-process significantly affects the mechanical properties of printouts. Washing removes the sol phase from the subsurface area of the sample. The rinsed voids can lead to the occurrenceof local stresses during post-curing. This may negatively affect the strength of the printouts. As the post-curing time increases, polymerization continues deeper inside the material. The result is a highly cross-linked polymer. Due to the optical permeability and light absorption by the dye (component of the resin), the samples may show local heterogeneity, caused by an inhomogeneous curing process intensity [5,11,12]. Both stages of the post-process—washing and post-curing—are significant, and their impact has not yet been satisfactorily described in the literature. The first publications have appeared only recently [11,12]. Analyzing the impact of the post-process on the properties of printouts, and expanding the knowledge base in this area, is highly purposeful.

The study concerns an attempt to estimate the influence of the time of the two stages of the post-process, as follows: washing and post-curing, on the mechanical properties of the obtained printouts, in particular on the strength. The study also attempted to identify the reasons for the change in printout strength after various post-process variants. For this purpose, the hardness was measured and the outer surface of the printouts was assessed with a profilographometer. Then, the correlation of these two physical factors with the strength results obtained in the static tensile and bending tests was assessed. The obtained results were analyzed in their entirety.

## 2. Materials and Methods

### 2.1. Printing and Postprocessing Procedure

**Figure 1:**
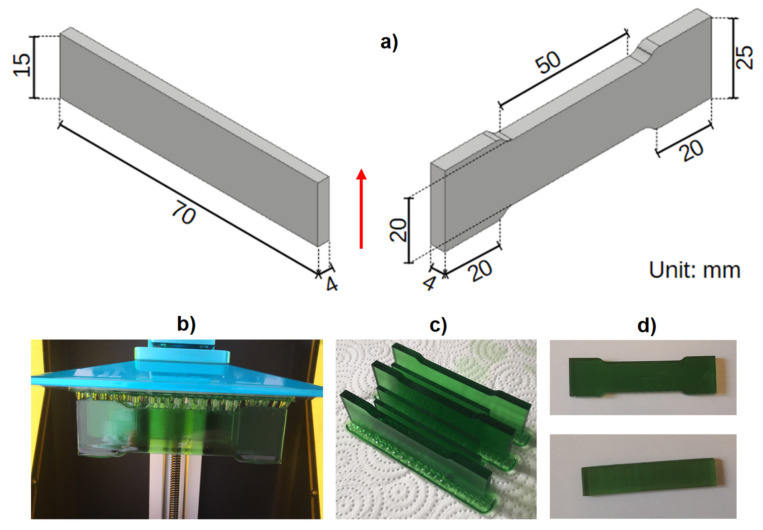
Printed specimens: (**a**) shape and dimensions, the red arrow shows the direction of the layer growth during printing, (**b**) the specimen after printing, (**c**) the specimens after post-process washing, (**d**) the specimens after post-curing.

The set of test samples was 3D-printed with the mSLA method using the Anycubic Photon printer (Shenzhen, China) [13,14]. Rectangular samples (for bending tests and for hardness testing and profilographometric evaluation) and paddle-shaped samples (for tensile tests) were printed. The shape and dimensions of the samples are shown in Figure 1.

**Figure 2:**
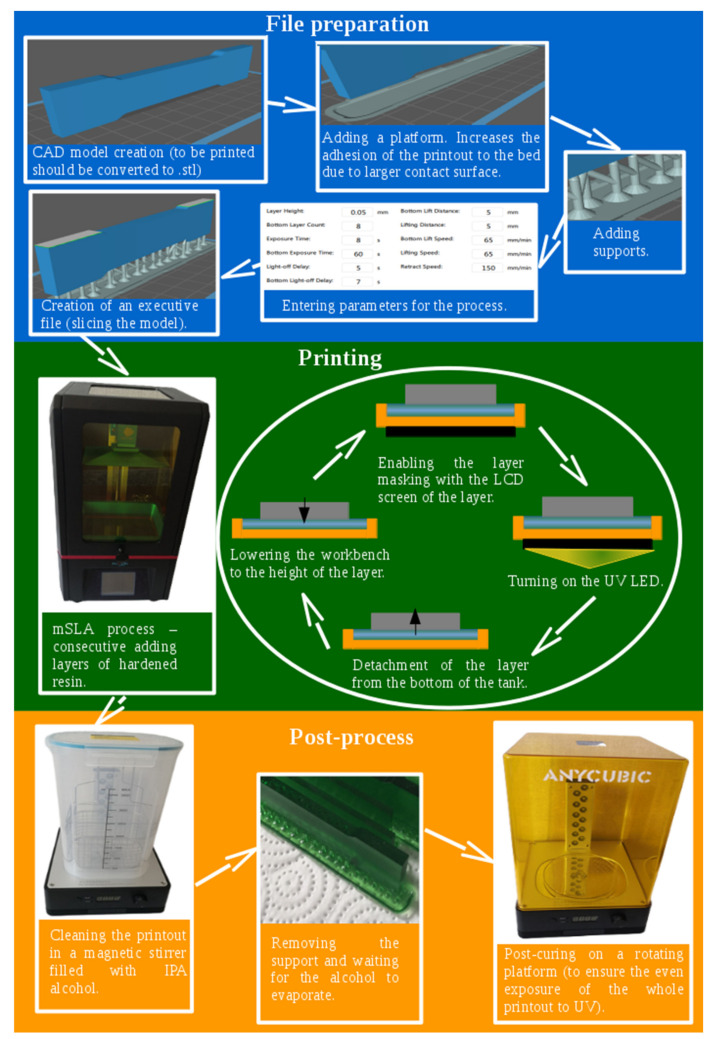
Graphical scheme of performed mSLA process.

The printing process was the same for all samples. Graphical scheme of the process is imaged in Figure 2. The samples were made of 3D printing UV-sensitive resin basic translucent green resin by Anycubic (Shenzhen, China). The samples were printed at an exposure time of 8 s for each layer 0.05 mm thick. The lift distance was 5 mm and the lifting speed was 65 mm/min. The speed of lowering the table is 150 mm/min. The resin vat temperature and the heatbed were 21–25 °C. The entire process was run at room temperature. The resin was not preheated. This ensured no defects in the sample resulting from the change in the temperature of the resin. For the tensile samples, the layers were arranged along the stretch direction. The layers were added along the wider side of the sample. For the bending samples, the layers were arranged parallel to the bend surface at an angle of 90°. The layers were added along the wider side of the sample. The samples were printed on supports that were 10 mm high. The tensile specimen was supported by 69 evenly spaced supports. The bending specimen was supported by 30 supports, also arranged evenly. The supports were arranged in a sequence of 2 on the outer parts of the lower wall of the sample and 1 in the middle following them. The printing resulted in “green” printouts with the assumed shape, but the consistency of a tough gel. Bringing to the final hardness required a post-process. The post-process consisted of the following two procedures: washing and post-curing. Both procedures were performed on an Anycubicwash &cure Machine 2.0 (Shenzhen, China) [15,16]. The washing procedure was carried out with 2-propanol, with the washed printout completely immersed. Washing time was different for each type of sample and amounted to 5, 10 or 30 min, which was to assess the effect of washing time on the properties of printouts. The post-curing procedure was carried under UV rays, with the longitudinal exposition of the printout and its uniform rotation around the longitudinal axis (theoretically—the entire surface was lit evenly).

As standard, all samples were exposed for 30 min. Only part of the samples previously washed for 10 min were intentionally exposed for 10 and 60 min, in order to assess the effect of exposure time on the properties of printouts. One series of samples was deliberately post-cured by being immersed in a glass container filled with water (1.5 mm glass, 30 mm water layer on each side). After post-process all samples were left at room temperature for a minimum of 3 days for acclimatization. They were then subjected to examination tests.

The manufacturer of the resin (and the printer) does not declare specific recommended washing time and post-curing time values in the technical documentation.It is recommended to wash briefly, but long enough to wash away any resin dripping from the sample.Then he recommends a long post-curing exposition. The maximum time of one exposition cycle is 60 min. Of course, after the end of the cycle, you can theoretically start another cycle many times. Most likely, according to the resin producer’s assumptions, the printing person himself must choose the time of the post-process procedures according to his own experience.

### 2.2. Examination Methods

The printed and post-processed samples have been put to the static tensile and static bending tests. The tensile tests were performed using INSTRON 4469 testing machine (INSTRON-CEAST, Norwood, MA, USA) [17], in accordance with the PN-EN ISO 527 standard, at the movement rate of 5 mm/min. The bending tests were carried out using ZWICK B2.5/TN1S machine (ZWICK-ROELL, Ulm, Germany) [18], in accordance with the PN-EN ISO 178 standard, at the rate of 10 mm/min. In each case, the stress obtained at a sample deflection of 6 mm was assumed as a flexural strength (R_g_). Five samples of each type were subjected to tensile and bending tests.

Separate set of samples (one of each type) was first subjected to profilographometric analysis using the MICRO PROF optical profilographometer by FRT (BergischGladbach, Germany) [19]. Then, they were subjected to Shore D hardness measurements using a SAUTER hardness tester (SAUTER, Wutoschingen, Germany) [20]. Hardness of outer surfaces of the samples was determined as average value after three measurements from one side and three from the opposite side. In order to measure the hardness in the inner area of a sample (central part of a cross-section), each sample was broken into two parts and the fracture surface was ground with abrasive paper (grain size in sequence: 600, 1000, 1600, 2000). Each time, the result was the average from six measurements in central line of the sample (equally between two outer surfaces).

Table 1 summarizes and describes all the sample types used in the study.

## 3. Results and Discussion

Table 2 and Table 3 summarize the results of the static tensile and bending tests.

**Figure 3:**
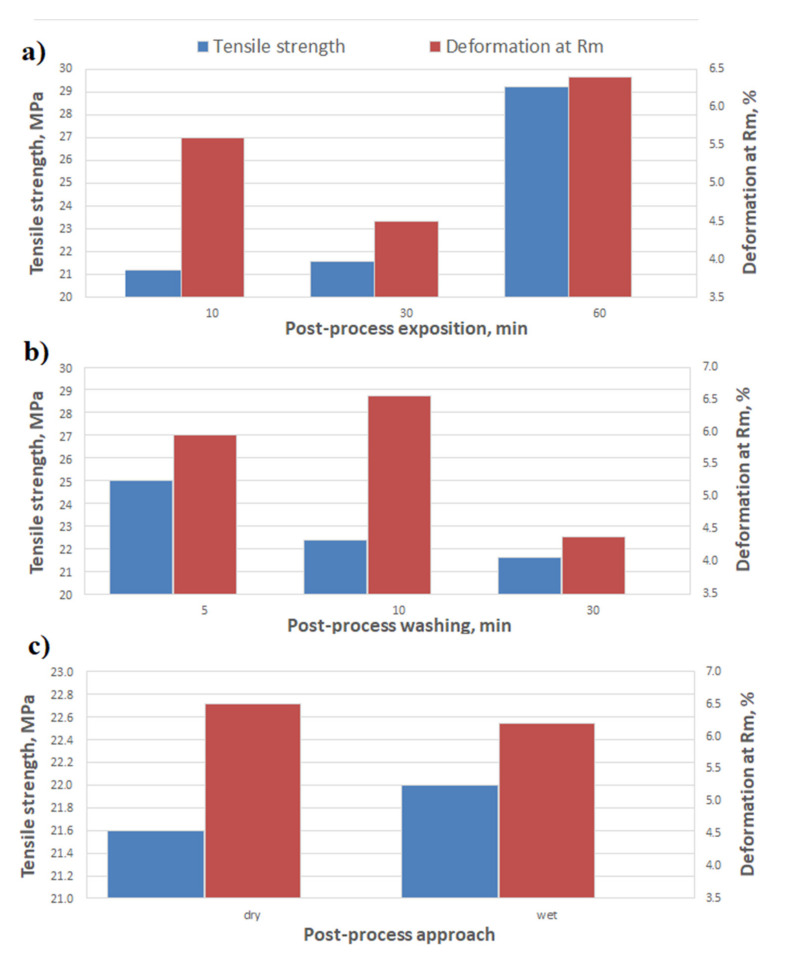
Tensile strength of printed samples, depending on the following: (**a**) post-curing exposition time, (**b**) post-process washing time, (**c**) post-process approach—post-curing in water shielding.

**Figure 4:**
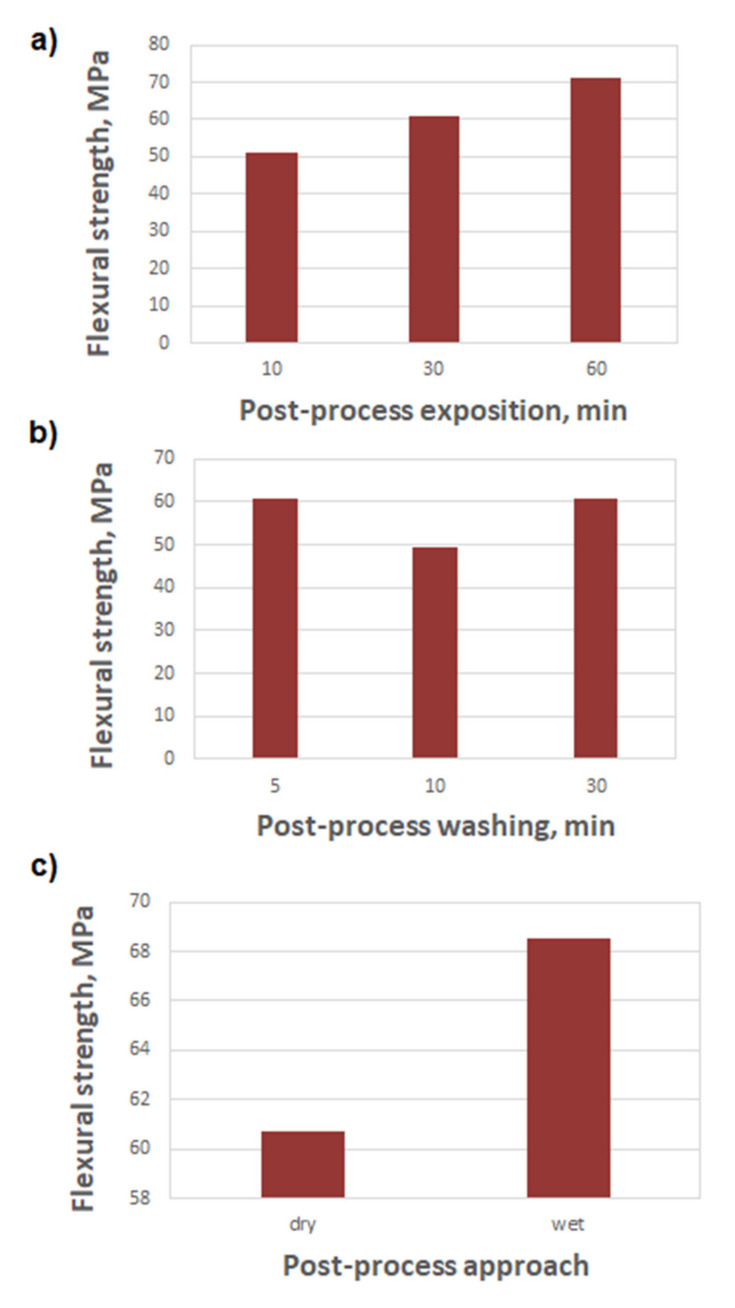
Flexural strength of printed samples, depending on the following: (**a**) post-curing exposition time, (**b**) post-process washing time, (**c**) post-process approach—post-curing in water shielding.

Figure 3 and Figure 4 show the results of the tensile and bending tests, compiled depending on the washing time and the post-curing time.

**Figure 5:**
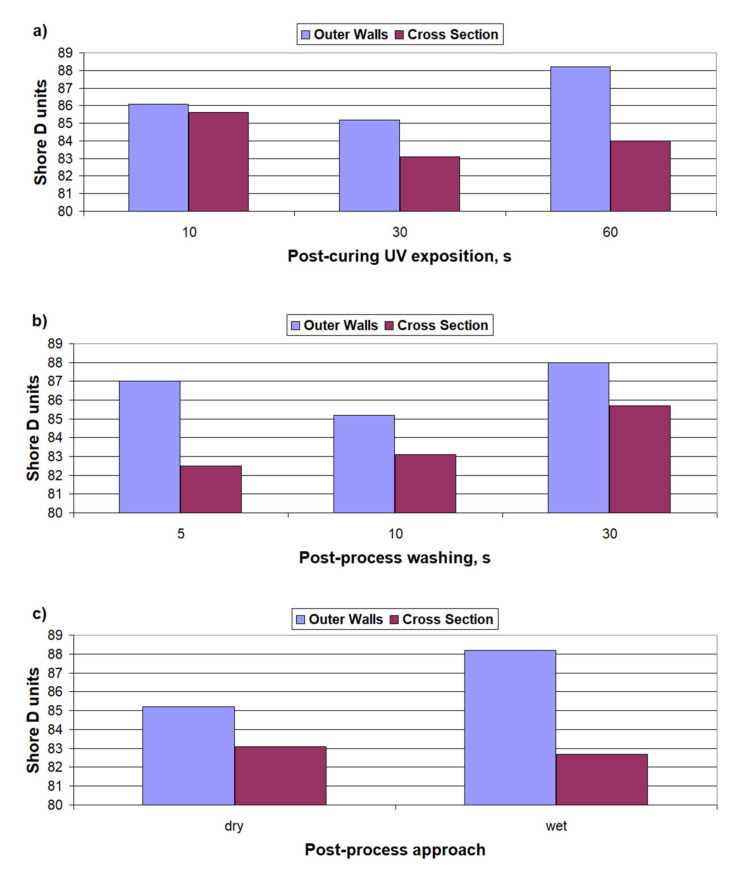
Shore D hardness of printed samples, depending on the following: (**a**) post-curing exposition time, (**b**) post-process washing time, (**c**) post-process approach—post-curing in water shielding.

Table 4 summarizes the results of the Shore hardness measurements (method D) of the produced samples. Figure 5 shows the hardness results compared to the washing time and to the post-curing time.

The obtained tensile and bending results (Figure 3a and Figure 4a) show clear the influence of the post-curing time on the strength of the samples. A significant increase in strength (especially tensile) at the longest exposure—by 38% in comparison to the shortest time—corresponds to an increase in the hardness (Figure 5a)—the increase is, respectively, 2.4%. It may result from the specific character of the resin curing process, which is both exothermic and thermally activated [3,21]. With a shorter exposure time, the energy supplied to the sample will not be able to heat the sample sufficiently to activate the polymerization in deeper areas, which exacerbates the energy shortage from the cross-linking reactions [22,23]. The final increase in the strain of the samples at R_m_, for a longer exposure time, is correlated with the increase in R_m_ (it is, respectively, 16% and 38%). This indicates an increase in the deformation capacity of the sample, mainly in the elastic range (this probably resulted from simple curing process progress for longer UV exposure). The effect of the washing time on the tensile and flexural strength (Figure 3b and Figure 4b) is slightly different. The tensile strength decreases with the increasing washing time, by 14%, and the flexural strength does not change at the end. In both the cases, the size of the variations are small. The decreased strength after a prolonged washing time may be due to the presence of more voids after sol removal from the subsurface area of the specimen, which may lead to a greater local stress concentration [24,25]. On the other hand, the reasons for the heterogeneity of R_m_ and R_g_ changes result from the different hardness in the inner and outer areas of the samples. The greater hardness of the outer walls and the lower hardness of the inner region of the sample that was post-cured with water shielding (wet) are observed in Figure 5c. The increase in the variation in the hardness between the subsurface areas and the areas inside the samples, caused by the post-curing time and by wet post-curing, may explain the larger increases in R_g_ as compared to the increases in R_m_. In the case of bending, the main loaded areas are the near-surface areas of the cross-section, and, in the case of stretching, the entire cross-section works evenly [26,27].

Post-curing in the water shielding had a particularly pronounced effect on the flexural strength of the printouts. This is probably related to the limitation of UV light access to the sample, and thus worse post-hardening of the internal areas. A worse cure means less hardness, by 3.5% (Figure 5c), which probably translates into increased plasticity of the material in the central part of the sample. This may hinder the development of the initiated failure cracks (overcoming plasticity through the crack is energy-consuming), especially caused by shear [28], and ultimately translates into an increase in flexural strength, by 38%.

The obtained hardness results indicate that both washing and post-curing do not have a decisive influence on the hardness of the resins. Both washing and post-curing cause a decrease in hardness (respectively, by 2% and 1%) first and, over a longer period of time, an increase (respectively, by 3.3% and 3.5%) in hardness in the area of the outer walls of the samples. The inner area of the samples shows strongly stochastic hardness changes. The observed results may result from the washing of excess resin from the printout (a process that is not very repeatable for different samples), which causes the absorbed energy to be used during the post-curing process, to cure a smaller amount of resin, which locally increases the specific density of this energy and increases the intensity of the process [3,4]. As a result, the effective degree of resin polymerization is greater, which translates into a greater hardness of the material. Most noticeable (and in line with expectations) is the increase in the hardness after wet post-curing. This fact is probably due to the lower oxygen content in the water than in the air. Oxygen slows down the polymerization process of the resin [29]. The limitation of its access has a major impact, as the results show—even greater than the limitation of UV rays to the sample, caused by the absorption by the layers of glass (container wall) and water. The wet post-cured samples show a simultaneous increase in the hardness of the walls (by 3.5%) and a decrease in the hardness of the inner area (by 0.5%). This is translated into the flexural strength, as described above, and as evidenced by the results in Figure 4c (38% increase in R_g_). This is confirmed by the aforementioned softening of a part of the sample cross-section, due to the reduced exposure of UV rays, resulting in incomplete curing of the resin.

**Figure 6:**
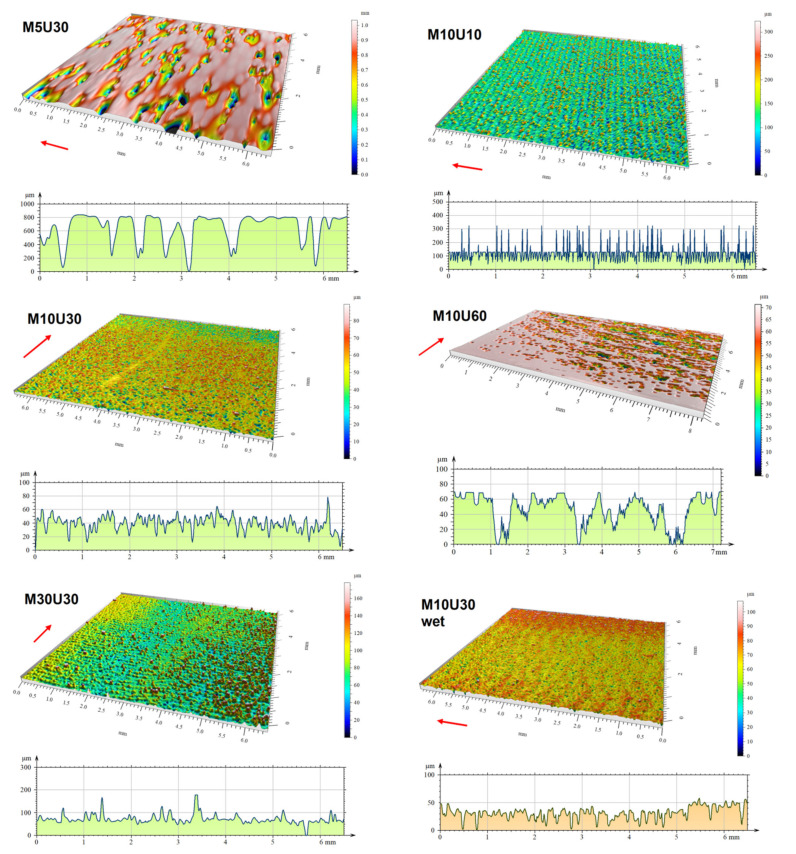
Digital images of the surface sections of individual sample types, made on an optical profilographometer, and representative profiles for each image. The red arrow indicates the direction of layer growth in the sample, which is also the direction of the profile.

Figure 6 shows digital images of the surface of the samples, taken on an optical profilographometer. The most representative surface profile is also shown for each image. The profile selection was automatic, carried out by the profilographometer software, for the best averaging of the measured surface parameters. The obtained surface images indicate that for a short time of washing (M5U30), we obtain the height of the profile (roughness) reaching the level of 1000 microns. This is a relatively huge value, and the shape of the profile indicates a large flattening, without finer serration. The profile is largely influenced by an excess of resin that is not removed during washing. A slight increase in the washing time (M10U30) causes a dramatic change in the surface and profile image. The large flat areas disappear, and serration appears, but with a much smaller depth—not exceeding 80 microns (basic height 50–70 microns). A serration of a width close to 50 microns (M10U10, M5U30) may result from the mapping of the print paths. In the case of the remaining samples (with longer washing times), the regularity of the serration is lost and the surface profile is most likely influenced by other factors, such as inhomogeneous washing out of the resin during washing, or inhomogeneous shrinkage of the resin in the subsurface area during post-curing. For the extended washing time (M30U30), we also observe a decrease in the density of the “teeth”—but their depth even increases. This is most likely due to the extrusion of uncured resin from the subsurface layers that comes out of the surface as spherical drops—this is visible in the image. For the M10U10 sample, we observe a significant washing effect on the profile height—it is a maximum of just over 30 microns, which is a much lower value than for the M5U30 sample, with half the washing time. At the same time, it can be observed that the extension of the exposure time in the post-curing (sample M10U30) reduces the profile height—perhaps it is related to the shrinkage of the resin during cross-linking. Further irradiation (sample M10U60) no longer leads to a reduction in the profile, but causes a reduction in the density of the “teeth”, this effect is also most likely due to the shrinkage of the resin crosslinking. However, there is a certain sequence of large flattenings, clearly separated by grooves approximately every 2 mm. It is possible that this is a representation of the sequence of the printing process after successfully curing (60 min of exposure) and shrinking all the residual resin. Introducing the sample into the water during the post-curing process (M10U30-wet) does not result in a significant change in the profile height or the overall surface image, but it causes unevenness (waviness) of the surface height on a larger scale—the image shows quite a significant difference in the profile height between the central area of the sample and the areas closer to the top and bottom edges.

There is a correlation between the obtained results of R_m_ and R_g_ and the imaged state of the surface. The washing effect on R_g_ may be due to a lower negative effect of the extracted droplets and a greater positive effect of the evening of the main base of the profile (see Figure 6, M30U30). The washing effect on R_m_, in turn, is counter-proportional—most likely diluting and removing too much uncured resin creates notch-like voids, which make the sample vulnerable to fast cracking during tensile loading. Longer post-curing has a positive effect on R_g_, but especially on R_m_—this is due to lowering the depth of the “teeth” (smaller notches) with prolonged exposure (compare M10U10 and M10U30/M10U60 in Figure 6). With wet post-curing, the profile is smoother and has no droplets (see M30U30 and M30U30-wet in Figure 6), clearly having a positive effect on R_g_, but not having a significant effect on R_m_. Probably, R_m_ depends mainly on the presence and depth of the notches, and these characteristics (number and depth of the “teeth”) are not significantly changed for wet post-curing in comparison with the “dry” process.

## 4. Conclusions

The samples that were produced by mask stereolithography were assessed for the effect of the post-process, divided into washing and post-curing, on mechanical properties and surface quality. The obtained results allowed us to draw the conclusions presented below.

(1) The following for the post-curing:
  - Post-curing time has a negative effect on the tensile strength and bending strength of the printouts.
  - Extending the post-curing time affects the heterogeneity of the produced printout. The hardness of the outer areas increases more than the area inside the printout. This does not have a negative effect on the strength, but contributes to a greater increase in the bending strength than in the tensile strength.
  - Post-curing improves the surface quality of the printouts. The improvement effect is visible up to a post-curing time of 30 min. Above this time, significant changes are no longer visible, but a large-scale sequencing of the surface relief appears.
(2) The following for the washing:
  - The obtained results indicate an ambiguous effect of washing on the strength of printouts. Finally, however, it should be said that prolonged washing slightly reduces the strength of the printed materials.
  - Longer washing times contribute to harder prints.
  - Washing significantly affects the quality of the printout surface. A washing time that is too short results in a surface that closely follows the sequencing characteristic of the printing process, with a roughness of several hundred microns. Increasing the washing time to 10 min lowers the roughness by one order of magnitude. Further increasing the washing time no longer has a positive effect on the surface relief of the printouts.
(3) The following for post-curing with water shielding:
  - Post-curing through the water layer causes a significant increase in hardness in the outer areas of the printouts, not affecting the hardness obtained in the inner areas. This translates into a higher flexural strength obtained by the samples post-cured through the water shielding.
  - The use of a water layer during post-curing does not affect the surface quality of the printout.
(4) The general conclusions are as follows:
  - The obtained mechanical test results and surface quality evaluation indicates that, for printouts with cross-sectional dimensions of several mm, the optimal post-process washing time is not more than 10 min, and the post-curing time is a minimum of 30 min.
  - The hardness results show that, already, at a depth of approx. 2 mm in relation to the outer surface, the hardness of the cured resin is clearly lower than its hardness on the printout surface. This differentiation progresses with post-curing time. This indicates that, from the point of view of mechanical properties, it is expedient to limit the thickness of the printed sections as much as possible, e.g., to design elements with unfilled cores.

## Figures and Tables

**Table 1:** Designations and descriptions of the samples used within the study.

Specimen Type Designation	Description (Post-Process Procedures Time)
M5U30	Specimen after 5 min washing and 30 min post-curing
M10U30	Specimen after 10 min washing and 30 min post-curing
M30U30	Specimen after 30 min washing and 30 min post-curing
M10U10	Specimen after 10 min washing and 10 min post-curing
M10U60	Specimen after 10 min washing and 60 min post-curing
M10U30-wet	Specimen after 10 min washing and 30 min post-curing, the samples were post-cured in a glass container with water.

**Table 2:** The results of static tensile tests of the produced printouts.

Specimen Type	Tensile Strength R_m_, MPa	Deformation at R_m_,%
M5U30	25.0(2.5)	5.8(0.7)
M10U30	22.4(2.6)	6.5(0.7)
M30U30	21.6(2.1)	4.0(0.3)
M10U10	21.2(3.2)	5.1(0.2)
M10U60	29.2(0.9)	5.9(0.6)
M10U30-wet	22.0(2.2)	6.2(0.9)

**Table 3:** The results of static bending tests of the produced printouts.

Specimen Type	Flexural Strength R_g_, MPa
M5U30	60.6(0.7)
M10U30	49.6(4.6)
M30U30	60.7(0.7)
M10U10	51.3(2.6)
M10U60	71.1(2.8)
M10U30-wet	68.5(1.2)

**Table 4:** Summary of hardness test (Shore method, type D) results for the produced printouts.

Specimen Type	Hardness (Shore D Units)	
	Outer Walls	Central Line of the Cross-Section
M5U30	87.0(0.4)	82.5(0.8)
M10U30	85.2(0.2)	83.1(0.5)
M30U30	88.0(0.4)	85.7(0.4)
M10U10	86.1(0.5)	85.6(0.6)
M10U60	88.2(0.4)	84.0(0.4)
M10U30-wet	88.2(0.4)	82.7(0.6)

## References

[1] Bartolo P.J. (2011). Stereolithography: Materials. Processes and Applications.

[2] Schmidleithner C., Kalaskar D.M. (2018). Stereolithography. 3D Print..

[3] Poth U., Schwalm R., Schwartz M., Baumstark R. (2011). Acrylic Resins.

[4] Penczek P., Królikowski W., Kłosowska-Wołkowicz Z. (2016). Nienasycone Żywice Poliestrowe.

[5] Ahmad K.W.H., Mohamad Z., Othman N., Che Man S.H., Jusoh M. (2020). He Mechanical Properties of Photopolymer Prepared Via 3D Stereolithography Printing: The Effect of UV Curing Time and Anisotropy. Chem. Eng. Trans..

[6] Erdogan B., Seyhan A., Ocak Y., Tanoglu M., Balkose D., Ulku S. (2008). Cure kinetics of epoxy resin-natural zeolite composites. J. Therm. Anal. Calorim..

[7] Minin A., Blatov I., Rodionov S., Zubarev I. (2021). Development of a cell co-cultivation system based on protein magnetic membranes, using a MSLA 3D printer. Bioprinting.

[8] Braszkiewicz M. Manufacturing of mechanical elements with properties of metamaterials using 3D printing technology. Proceedings of the 34th Scientific Conference: Problems of Working Machines Development.

[9] Kumar S., Bhushan P., Pandey M., Bhattacharya S. (2019). Additive manufacturing as an emerging technology for fabrication of microelectromechanical systems (MEMS). J. Micromanufac..

[10] Esposito L., Sorrentino L., Penta F., Bellini C. (2016). Effect of curing overheating on interlaminar shear strength and its modelling in thick FRP laminates. Int. J. Adv. Manuf. Technol..

[11] Miedzinska D., Gieleta R., Popławski A. (2020). Experimental study on influence of curing time on strength behavior of SLA-printed samples loaded with different strain rates. Materials.

[12] Pszczółkowski B., Dzadz Ł. (2020). Analysis of the influence of UV light exposure time on hardness and density properties of SLA models. Tech. Sci..

[13] https://www.anycubic.com/collections/anycubic-photon-3d-printers/products/anycubic-photon-3d-printer.

[14] https://drive.google.com/file/d/1gGvgaVlVSFrv6Ad0xq8NZv_crqn2u_rz/view.

[15] https://www.anycubic.com/products/wash-cure-machine-2-0.

[16] https://cdn-reichelt.de/documents/datenblatt/E910/ANYCUBIC_WASHANDCURE_2_ANL-EN.pdf.

[17] https://www.eng.uc.edu/~beaucag/Classes/Characterization/M10-24400-1-2%20%284465%20manual%29.pdf.

[18] https://www.zwickroell.com/products/static-materials-testing-machines/universal-testing-machines-for-static-applications/zwickiline/.

[19] https://www.iveindhoven.com/en/article/frt-microprof-optical-profilometer-surfacemeter/.

[20] https://www.sauter.eu/shop/en/measuring-instruments/hardness-testing-of-plastics-shore-/HB/.

[21] Chatys R., Piernik K. (2021). Influence pf speed of resin injection under pressure into mould on strength properties of polymer composite. Compos. Theory Pract..

[22] Oliwa R., Heneczkowski M., Oleksy M., Galina H. (2016). Epoxy composites of reduced flammability. Compos. Part B Eng..

[23] Oleksy M., Szwarc-Rzepka K., Heneczkowski M., Oliwa R., Jesionowski T. (2014). Epoxy resin composite based on functional hybrid fillers. Materials.

[24] Sikorski R. (2021). Macro- and micromechanical response of glass fibre-reinforced polypropylene to pigmental impurities. Compos. Theory Pract..

[25] Seyhan A.T., Sun Z., Deitzel J., Tanoglu M., Heider D. (2009). Cure kinetics of vapor grown carbon nanofiber (VGCNF) modified epoxy resin suspensions and fracture toughness of their resulting nanocomposites. Mater. Chem. Phys..

[26] Okrajni J. (2002). Podstawy Mechaniki Technicznej dla Materiałoznawców.

[27] Alam S., Chowdhury M.A. (2020). Micromechanical analysis of glass fiber reinforced epoxy composites and case study of macro-mechanical observation. Compos. Theory Pract..

[28] Ganczarski A., Skrzypek J. (2009). Plastyczność Materiałów Inżynierskich.

[29] Studer K., Decker C., Beck E., Schwalm R. (2003). Overcoming Oxygen Inhibition in UV-Curing of Acrylate Coatings by Carbon Dioxide Inerting. Prog. Org. Coat..

